# Nonlinear Buckling Behavior of Spiral Corrugated Sandwich FGM Cylindrical Shells Surrounded by an Elastic Medium

**DOI:** 10.3390/ma13081984

**Published:** 2020-04-24

**Authors:** Vu Tho Hung, Dang Thuy Dong, Nguyen Thi Phuong, Le Ngoc Ly, Tran Quang Minh, Nguyen-Thoi Trung, Vu Hoai Nam

**Affiliations:** 1 Faculty of Civil Engineering, University of Transport Technology, Hanoi 100000, Vietnam; hungvt@utt.edu.vn (V.T.H.); dongdt@utt.edu.vn (D.T.D.); phuongnt@utt.edu.vn (N.T.P.); minhtq@utt.edu.vn (T.Q.M.); 2 Faculty of Fundamental Science for Engineering, University of Transport Technology, Hanoi 100000, Vietnam; lyln@utt.edu.vn; 3 Division of Computational Mathematics and Engineering, Institute for Computational Science, Ton Duc Thang University, Ho Chi Minh City 700000, Vietnam; nguyenthoitrung@tdtu.edu.vn; 4 Faculty of Civil Engineering, Ton Duc Thang University, Ho Chi Minh City 700000, Vietnam

**Keywords:** sandwich FGM, spiral corrugated cylindrical shells, postbuckling behavior, external pressure, semi-analytical approach

## Abstract

This paper presents a semi-analytical approach for investigating the nonlinear buckling and postbuckling of spiral corrugated sandwich functionally graded (FGM) cylindrical shells under external pressure and surrounded by a two-parameter elastic foundation based on Donnell shell theory. The improved homogenization theory for the spiral corrugated FGM structure is applied and the geometrical nonlinearity in a von Karman sense is taken into account. The nonlinear equilibrium equation system can be solved by using the Galerkin method with the three-term solution form of deflection. An explicit solution form for the nonlinear buckling behavior of shells is obtained. The critical buckling pressure and the postbuckling strength of shells are numerically investigated. Additionally, the effects of spiral corrugation in enhancing the nonlinear buckling behavior of spiral corrugated sandwich FGM cylindrical shells are validated and discussed.

## 1. Introduction

Due to their excellent thermo-mechanical properties, functionally graded materials (FGMs) have been applied in engineering structures, such as military structures, nuclear reactors, civil engineering structures, and other technology areas [1]. Research on the thermo-mechanical behavior of FGM cylindrical plates and shells has been the focus of researchers across the world in recent decades.

Based on Love’s shell theory, Arshad et al. [2,3,4] studied the linear frequency of the free vibration of FGM and two-layer FGM thin cylindrical shells with algebraic polynomial, exponential, and trigonometric distribution laws of material by using the Rayleigh–Ritz method [2,3] and wave propagation approach [4]. Shen et al. [5] studied the nonlinear postbuckling of shear deformable pressure loaded FGM cylindrical shells with the shell-foundation interaction effect. Furthermore, Ebrahimi and Sepiani [6] compared the linear buckling and natural frequency of static and period loaded FGM cylindrical shells for the results of classical thin shell theory and first-order shear deformation theory (FSDT). Additionally, Isvandzibaei et al. [7,8,9] investigated the free vibration behavior of FGM cylindrical shells with ring support effects based on Love–Kirchhoff [7] and first-order theory [8,9], with various boundary conditions. Kim [10] investigated the free vibration analysis of shear deformable FGM cylindrical shells partially surrounded by a Pasternak foundation model with an oblique edge. Moreover, Sun et al. [11] presented an analytical approach for investigating the linear buckling of higher-order shear deformable FGM cylindrical shells subjected to axial compressive and thermal loads. The nonlinear vibration behavior and dynamic buckling of FGM cylindrical shells with and without three-parameter elastic foundation were investigated using Donnell shell theory with von Karman nonlinearity [12,13]. Additionally, Thai et al. [14] discussed the porosity effects of FGM conical shells reinforced by eccentric FGM stiffeners using an analytical approach. By employing the higher-order shear deformation theory, Vuong and Duc [15] investigated the nonlinear buckling of FGM toroidal shell segments and cylindrical shells reinforced by orthogonal stiffeners with temperature effects. Furthermore, Dung and Hoa [16] studied the nonlinear buckling of FGM cylindrical shells reinforced by FGM stiffeners in longitudinal and circumferential directions and subjected to radial loads. By improving the smeared stiffener technique for spiral stiffeners, the nonlinear thermomechanical buckling and postbuckling behavior of FGM, sandwich FGM, and multilayer FGM cylindrical shells stiffened by FGM or isotropic spiral stiffeners subjected to various loading conditions was studied by Nam et al. [17,18,19] and Phuong et al. [20,21]. Based on the FSDT, Esmaeili et al. [22] discussed the nonlinear axisymmetric vibration behavior of shear deformable FGM cylindrical shells under rapid heating on the ceramic-rich surface, while a constant temperature was applied for the metal-rich surface. Sofiyev and Hui [23] developed an analytical approach for linear vibration responses of shear deformable FGM cylindrical shells with mixed boundary conditions to obtain the closed-form solutions of a problem. Moreover, the linear free vibration behavior of thick or moderately thick FGM circular cylindrical shells with a Pasternak elastic foundation and shell-fluid interaction effects was investigated by Shahbaztabar et al. [24] and Baghlani et al. [25], respectively. The vibration behavior of stepped FGM paraboloidal shells, taking into account the general edge constraints, was mentioned by Pang et al. [26].

For corrugated cylindrical shells, some previous studies have focused on experimental and numerical analyses of the mechanical behavior of these structures. Yang et al. [27] established governing equations and proposed a numerical approximation of the nonlinear deformation problem of longitudinally corrugated cylindrical shells under a uniformly distributed load. Ghazijahani et al. [28] presented experimental results on the buckling behavior of corrugated cylindrical shells and cylindrical shells stiffened by corrugated rings under a uniform peripheral pressure. Ahmed [29] developed a simply analytical approach for investigating the linear buckling behavior of a cosine corrugated elliptic cylindrical shell subjected to external pressure and surrounded by an elastic foundation. Additionally, Xiong et al. [30] presented a fabrication process and experimental results of mechanical responses of carbon fiber composite core-corrugated sandwich cylindrical shells. Iwicki et al. [31,32] and Hajko et al. [33] presented an economic design and proposed a numerical model for analyzing the linear global buckling behavior of corrugated silos stiffened by longitudinal stiffeners. Furthermore, Su et al. [34] presented experimental and linear analytical results on the collapse behavior of core-corrugated sandwich cylindrical shells subjected to axial compression. Yang et al. [35,36] presented experimental and numerical results on the vibration and low velocity impact behavior of composite core-corrugated sandwich cylindrical shells and panels.

Due to their excellent load carrying capacity and conformance with manufacturing technology, spiral corrugated cylindrical shells are often used in engineering structures. According to the best of the authors’ knowledge, there is currently no semi-analytical approach for investigating the mechanical behavior of spiral corrugated cylindrical shells made of sandwich FGM surrounded by an elastic foundation. This paper presents a semi-analytical approach for examining the nonlinear buckling responses of spiral corrugated sandwich FGM circular cylindrical shells using a homogenization technique (Xia et al. [37]). The spiral corrugation is homogenized by combining a homogenization model and the coordinate transformation procedure. Based on Donnell shell theory with nonlinear terms in the von Karman sense, the three-term solution of deflection, and the Galerkin method, a stability equation system is obtained. The effects of corrugation and the material distribution on the nonlinear buckling responses are investigated using the numerical results.

## 2. Homogenization Process of Spiral Corrugated Sandwich FGM Cylindrical Shells

**Figure 1:**
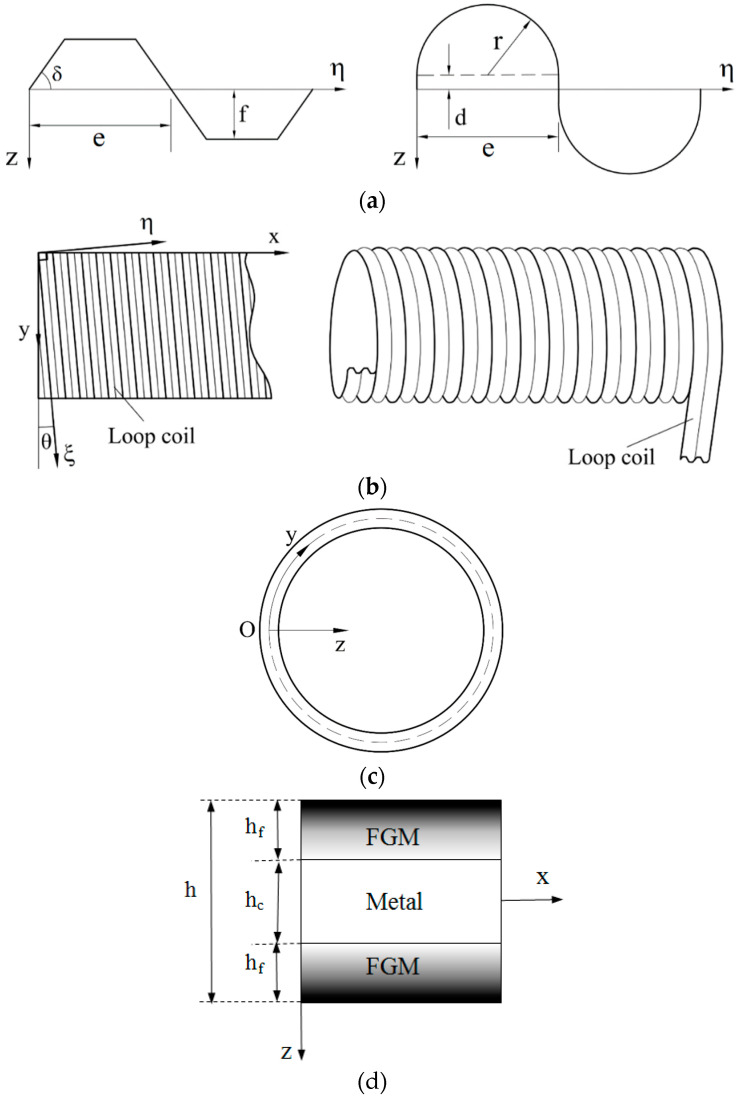
Configurations, local and global coordinate systems, and the material distribution of sandwich functionally graded material (FGM) cylindrical shells and corrugations. (**a**) Cross section of trapezoidal and round corrugations; (**b**) local coordinate systems; (**c**) global coordinate systems; (**d**) distribution law of sandwich FGM.

Consider a spiral corrugated sandwich FGM cylindrical shell with an axial length L, thickness h, and circumferential radius R. According to the manufacturing process, the spiral corrugated cylindrical shell is made by coiling corrugated material. Corrugations can be classified as two types: round and trapezoidal corrugations. The geometrical properties of the cross section of corrugations is defined by e,f,δ for trapezoidal corrugations and e,r,d for round corrugations, as shown in Figure 1a.

Based on the geometrical calculations, the relation between the angle of corrugation and the number of full waves of corrugation in a loop coil can be established as

(1) θ=arcsin(Ne/πR)

where the number of full waves of corrugation in a loop coil is denoted by N.

The geometrical parameters of the cross section of corrugations (r,d,e,f) are chosen so that they must smaller than the global parameters (R,L) of the shell. This assumption is an important condition which allows the application of Xia et al.’s homogenization model [37] for corrugated shells.

The considered corrugated shell is exposed in the global coordinate system Oxyz, with x as the longitudinal axis, y=Rθ as the circumferential axis, and z as the inward normal axis (Figure 1b,c). The elastic medium is modeled by using Pasternak’s elastic foundation model with two elastic moduli: $K_{1}$ and $K_{2}$. The volume fractions of the ceramic and metal of the external layer are continuously varied through the thickness of the shell, from a full ceramic surface (z=−h/2) to a full metal surface ($z=-h/2+h_{f}$), and the volume fractions of the ceramic and metal of the internal layer are continuously varied through the thickness, from a full metal surface ($z=h/2-h_{f}$) to a full ceramic surface (z=h/2), and the full metal core layer is used (see Figure 1d).

The elastic modulus E(z) of the shell is assumed to be the extended Sigmoid power law:

(2) $$\left[E\left(z\right)\right]=\left\{ \begin{array}{ll} \left[E_{c}\right]+\left[E_{m}-E_{c}\right]{\left(\frac{2z+h}{2h_{f}}\right)}^{k}, & -\frac{h}{2}\leq z\leq -\frac{h}{2}+h_{f},\\ \left[E_{m}\right], & -\frac{h}{2}+h_{f}\;\leq z\leq \frac{h}{2}-h_{f},\\ \left[E_{c}\right]+\left[E_{m}-E_{c}\right]{\left(\frac{-2z+h}{2h_{f}}\right)}^{k}, & \frac{h}{2}-h_{f}\leq z\leq \frac{h}{2}, \end{array}\right.$$

where k is the volume fraction index and the elastic moduli of the metal and ceramic are denoted by $E_{m}$ and $E_{c}$, respectively.

Stress–strain relations for a non-corrugated sandwich FGM shell are expressed as (in the local coordinate system)

(3) $$\left(\begin{array}{c} \sigma_{\eta }\\ \sigma_{\xi }\\ \sigma_{\eta \xi } \end{array}\right)=E\left(z\right)\left(\begin{array}{ccc} \frac{1}{1-\nu^{2}} & \frac{\nu }{1-\nu^{2}} & 0\\ \frac{\nu }{1-\nu^{2}} & \frac{1}{1-\nu^{2}} & 0\\ 0 & 0 & \frac{1}{2\left(1+\nu \right)} \end{array}\right)\left\{\begin{array}{c} \varepsilon_{\eta }\\ \varepsilon_{\xi }\\ \gamma_{\eta \xi } \end{array}\right\},$$

where the Poisson ratio ν is assumed to be a constant value.

The force and moment results of a sandwich cylindrical shell (in the local coordinate system) are expressed by applying the homogenization procedure for corrugated structures established by Xia et al. [37], as

(4) $$\left\{\begin{array}{l} N_{\eta }\\ N_{\xi }\\ N_{\eta \xi }\\ M_{\eta }\\ M_{\xi }\\ M_{\eta \xi } \end{array}\right\}=\left\{\begin{array}{cccccc} X_{11} & X_{12} & 0 & 0 & 0 & 0\\ X_{12} & X_{22} & 0 & 0 & 0 & 0\\ 0 & 0 & X_{66} & 0 & 0 & 0\\ 0 & 0 & 0 & Y_{11} & Y_{12} & 0\\ 0 & 0 & 0 & Y_{12} & Y_{22} & 0\\ 0 & 0 & 0 & 0 & 0 & Y_{66} \end{array}\right\}\left\{\begin{array}{l} \varepsilon_{\eta }^{0}\\ \varepsilon_{\xi }^{0}\\ \gamma_{\eta \xi }^{0}\\ \chi_{\eta }\\ \chi_{\xi }\\ 2\chi_{\eta \xi } \end{array}\right\},$$

where the changes of curvatures and twists are defined as

$$\chi_{\eta }=-\frac{\partial^{2}w}{\partial x^{2}},\chi_{\xi }=-\frac{\partial^{2}w}{\partial y^{2}},\chi_{\eta \xi }=-\frac{\partial^{2}w}{\partial x\partial y}$$

and the stiffness matrix terms of Equation (4) are expressed as

$$X_{11}=\frac{2eF_{11}G_{11}}{I_{1}G_{11}+I_{2}F_{11}},\;\;\;\;X_{12}=\frac{F_{12}}{F_{11}}X_{11},\;\;\;\;X_{22}=\frac{X_{12}F_{12}}{F_{11}}+\frac{l}{e}\frac{F_{11}F_{22}-F_{12}^{2}}{F_{11}},\;\;\;\;X_{66}=\frac{e}{l}F_{66},\;Y_{11}=\frac{eG_{11}}{l},\;\;\;\;\;\;\;\;\;\;\;\;\;\;\;\;Y_{12}=\frac{G_{12}Y_{11}}{G_{11}},\;\;\;\;\;\;\;\;\;\;\;\;\;\;\;\;\;\;Y_{22}=\frac{I_{2}F_{22}+I_{1}G_{22}}{2e},\;\;\;\;\;\;\;\;\;\;\;\;\;\;\;\;\;\;Y_{66}=\frac{lG_{66}}{e},$$

in which, for round corrugations,

$$l=\pi r+2d,\;e=2r,\;I_{1}=\pi r,\;\;\;I_{2}=\frac{4d^{3}}{3}+2\pi d^{2}r+8dr^{2}+\pi r3$$

and for trapezoidal corrugations,

$$l=\frac{2f}{\sin\delta }+e-\frac{2f}{\tan\delta },\;I_{1}=\frac{4f\cos\delta }{3\sin\delta }+2e-\frac{4f}{\tan\delta },\;I_{2}=\frac{4f^{3}}{3\sin\delta }+2f^{2}\left(e-\frac{2f}{\tan\delta }\right).$$

The stiffness matrix terms of non-corrugated shells can be determined as

(5) $$\begin{array}{l} F_{11}=F_{22}=\frac{C_{1}}{1-\nu^{2}},\;\;\;\;\;\;F_{12}=\frac{\nu C_{1}}{1-\nu^{2}},\;\;\;\;\;\;F_{66}=\frac{C_{1}}{2\left(1-\nu \right)},\\ G_{11}=G_{22}=\frac{C_{2}}{1-\nu^{2}},\;\;\;\;\;\;G_{12}=\frac{\nu C_{2}}{1-\nu^{2}},\;\;\;\;\;\;G_{66}=\frac{C_{2}}{2\left(1-\nu \right)},\\ C_{1}=E_{c}h+\left(E_{m}-E_{c}\right)\left(h_{c}+2\frac{h_{f}}{k+1}\right),\\ C_{2}=E_{c}\frac{h^{3}}{12}+\left(E_{m}-E_{c}\right)\left[\frac{h^{2}h_{f}}{2\left(k+1\right)}-\frac{2hh_{f}^{2}}{k+2}+\frac{2h_{f}^{3}}{k+3}+\frac{{\left(h-2h_{f}\right)}^{3}}{12}\right]. \end{array}$$

Based on the coordinate transformation technique, the force and moment results of the corrugated sandwich FGM shells in the (Oxyz) system can be obtained as

(6) $$\left\{\begin{array}{c} N_{x}\\ N_{y}\\ N_{xy}\\ M_{x}\\ M_{y}\\ M_{xy} \end{array}\right\}=\left\{\begin{array}{cccccc} A_{11} & A_{12} & A_{16} & 0 & 0 & 0\\ A_{12} & A_{22} & A_{26} & 0 & 0 & 0\\ A_{16} & A_{26} & A_{66} & 0 & 0 & 0\\ 0 & 0 & 0 & D_{11} & D_{12} & D_{16}\\ 0 & 0 & 0 & D_{12} & D_{22} & D_{26}\\ 0 & 0 & 0 & D_{16} & D_{26} & D_{66} \end{array}\right\}\left\{\begin{array}{c} \varepsilon_{x}^{0}\\ \varepsilon_{y}^{0}\\ \gamma_{xy}^{0}\\ \chi_{x}\\ \chi_{y}\\ 2\chi_{xy} \end{array}\right\},$$

where

(7) $$\left[\begin{array}{c} A_{11}\\ A_{12}\\ A_{22}\\ A_{16}\\ A_{26}\\ A_{66} \end{array}\right]=\left[\begin{array}{cccc} c^{4} & 2c^{2}s^{2} & s^{4} & 4c^{2}s^{2}\\ c^{2}s^{2} & c^{4}+s^{4} & c^{2}s^{2} & -4c^{2}s^{2}\\ s^{4} & 2c^{2}s^{2} & c^{4} & 4c^{2}s^{2}\\ c^{3}s & cs^{3}-c^{3}s & -cs^{3} & -2cs\left(c^{2}-s^{2}\right)\\ cs^{3} & c^{3}s-cs^{3} & -c^{3}s & 2cs\left(c^{2}-s^{2}\right)\\ c^{2}s^{2} & -2c^{2}s^{2} & c^{2}s^{2} & {\left(c^{2}-s^{2}\right)}^{2} \end{array}\right]\left[\begin{array}{c} X_{11}\\ X_{12}\\ X_{22}\\ X_{66} \end{array}\right],$$

(8) $$\left[\begin{array}{c} D_{11}\\ D_{12}\\ D_{22}\\ D_{16}\\ D_{26}\\ D_{66} \end{array}\right]=\left[\begin{array}{cccc} c^{4} & 2c^{2}s^{2} & s^{4} & 4c^{2}s^{2}\\ c^{2}s^{2} & c^{4}+s^{4} & c^{2}s^{2} & -4c^{2}s^{2}\\ s^{4} & 2c^{2}s^{2} & c^{4} & 4c^{2}s^{2}\\ c^{3}s & cs^{3}-c^{3}s & -cs^{3} & -2cs\left(c^{2}-s^{2}\right)\\ cs^{3} & c^{3}s-cs^{3} & -c^{3}s & 2cs\left(c^{2}-s^{2}\right)\\ c^{2}s^{2} & -2c^{2}s^{2} & c^{2}s^{2} & {\left(c^{2}-s^{2}\right)}^{2} \end{array}\right]\left[\begin{array}{c} Y_{11}\\ Y_{12}\\ Y_{22}\\ Y_{66} \end{array}\right],$$

in which c=cosθ, and s=sinθ.

As can be recognized, the spiral corrugated sandwich FGM cylindrical shells are equivalent to the non-corrugated sandwich FGM cylindrical shells with the new stiffness matrix presented in Equation (6).

## 3. Stability Equation Establishment

Based on the nonlinear strain–displacement assumption of von Karman and Donnell shell theory, the strains at the mid-plane of non-corrugated sandwich FGM cylindrical shells can be expressed as [38]

(9) $$\begin{array}{l} \varepsilon_{x}^{0}=u_{,x}+\frac{{\left(w_{,x}\right)}^{2}}{2},\\ \varepsilon_{y}^{0}=v_{,y}-\frac{w}{R}+\frac{{\left(w_{,y}\right)}^{2}}{2},\\ \gamma_{xy}^{0}=u_{,y}+v_{,x}+w_{,x}w_{,y}, \end{array}$$

where $\varepsilon_{x}^{0}$ and $\varepsilon_{y}^{0}$ are the normal strains; $\gamma_{xy}^{0}$ is the shear strain at the mid-plane; and u, v, and w are displacements in x, y, and z directions of the cylindrical shell, respectively.

The strains across the shell thickness at a distance z from the mid-plane are represented by

(10) $$\varepsilon_{x}=\varepsilon_{x}^{0}-zw_{,xx},\;\;\;\;\;\;\;\;\;\;\;\;\varepsilon_{y}=\varepsilon_{y}^{0}-zw_{,yy},\;\;\;\;\;\;\;\;\;\;\;\;\gamma_{xy}=\gamma_{xy}^{0}-2zw_{,xy}.$$

From Equation (9), the deformation compatibility equation can be directly established as

(11) $$\varepsilon_{x,yy}^{0}+\varepsilon_{y,xx}^{0}-\gamma_{xy,xy}^{0}=w_{,xy}^{2}-w_{,xx}w_{,xy}-\frac{1}{R}w_{,xx}.$$

The nonlinear equilibrium equations of a non-corrugated cylindrical shell based on Donnell shell theory are

(12) $$\begin{array}{l} N_{x,x}+N_{xy,y}=0,\;\;\;\;\;\;\;\;\;N_{y,y}+N_{xy,x}=0,\\ M_{x,xx}+2M_{xy,xy}+M_{y,yy}+\frac{N_{y}}{R}+N_{x}w_{,xx}+2N_{xy}w_{,xy}\\ \;\;\;\;\;\;\;\;\;\;\;\;\;\;\;\;\;+N_{y}w_{,yy}=-q_{0}+K_{1}w-K_{2}\left(w_{,xx}+w_{,yy}\right). \end{array}$$

Considering the first two equations of Equation (12), a stress function ϕ can be introduced, which satisfies the following conditions:

(13) $$N_{x}=\phi_{,yy},N_{y}=\phi_{,xx},N_{xy}=-\phi_{,xy}.$$

Substituting Equations (6) and (13) into the third equation of (12), the nonlinear equilibrium equation is obtained in a new form, as

(14) $$\begin{array}{l} w_{,xxxx}D_{11}+w_{,xxxy}4D_{16}+w_{,xxyy}\left(2D_{12}+4D_{66}\right)\\ \;\;\;\;\;\;\;\;+w_{,xyyy}4D_{26}+w_{,yyyy}D_{22}-\phi_{,yy}w_{,xx}-\phi_{,xx}w_{,yy}\\ \;\;\;\;\;\;\;\;\;\;\;\;\;\;\;\;\;\;\;\;\;+2\phi_{,xy}w_{,xy}-\frac{\phi_{,xx}}{R}=q_{0}-K_{1}w+K_{2}\left(w_{,xx}+w_{,yy}\right). \end{array}$$

From the constitutive relations of Equation (6), the inverse relations can be established, taking into account Equation (13), as

(15) $$\left\{\begin{array}{c} \varepsilon_{x}^{0}\\ \varepsilon_{y}^{0}\\ \gamma_{xy}^{0} \end{array}\right\}=\left\{\begin{array}{ccc} H_{11} & H_{12} & H_{16}\\ H_{12} & H_{22} & H_{26}\\ H_{16} & H_{26} & H_{66} \end{array}\right\}\left\{\begin{array}{c} \phi_{,yy}\\ \phi_{,xx}\\ -\phi_{,xy} \end{array}\right\},$$

where

$$H_{11}=\frac{A_{22}A_{66}-A_{62}A_{26}}{\Omega },H_{12}=\frac{A_{61}A_{26}-A_{21}A_{66}}{\Omega },H_{16}=\frac{A_{21}A_{62}-A_{61}A_{22}}{\Omega },H_{22}=\frac{A_{11}A_{66}-A_{61}A_{16}}{\Omega },H_{26}=\frac{A_{61}A_{12}-A_{11}A_{62}}{\Omega },H_{66}=\frac{A_{11}A_{22}-A_{21}A_{12}}{\Omega },\Omega =\left(A_{11}A_{22}A_{66}+2A_{12}A_{26}A_{16}\right)-\left(A_{16}^{2}A_{22}+A_{12}^{2}A_{66}+A_{11}A_{26}^{2}\right).$$

By substituting Equation (15) into the deformation compatibility Equation (11), the equation can be rewritten as

(16) $$\begin{array}{l} \phi_{,xxxx}H_{22}-\phi_{,xxxy}2H_{26}+\phi_{,xxyy}\left(2H_{12}+H_{66}\right)\\ \;\;\;\;\;\;\;\;\;\;\;-\phi_{,xyyy}2H_{16}+\phi_{,yyyy}H_{11}+\frac{1}{R}w_{,xx}-w_{,xy}^{2}+w_{,xx}w_{,xy}=0. \end{array}$$

## 4. Three-Term Solution and Galerkin Procedure

Consider a spiral corrugated sandwich FGM cylindrical shell under external pressure $q_{0}$. In this case, the simply supported boundary condition has the following form:

(17) $$N_{x}=0,\;N_{xy}=0,\;M_{x}=0,\;w=0,\;at\;x=0;\;L.$$

The solution of the deflection satisfying the boundary condition in Equation (17) in an approximate sense can be applied as a three-state form:

(18) $$w=f_{0}+f_{1}\sin\frac{m\pi x}{L}\sin\frac{ny}{R}+f_{2}\sin^{2}\frac{m\pi x}{L},$$

in which $f_{0}$ is the amplitude of the uniform pre-buckling state, $f_{1}$ is the amplitude of the linear postbuckling state, $f_{2}$ is the amplitude of the nonlinear postbuckling state, the linear buckling shape is modeled by the term $\sin\frac{m\pi x}{L}\sin\frac{ny}{R}$, the nonlinear buckling shape on the longitudinal axis is modeled by the term $\sin^{2}\frac{m\pi x}{L}$, m is the number of half waves on the longitudinal axis, and n is the number of half waves on the circumferential axis.

Clearly, the simply supported boundary conditions in Equation (17) are approximately satisfied.

Substituting Equation (18) into Equation (16), the form of the stress function ϕ can be obtained as

(19) $$\begin{array}{l} \phi =\phi_{1}\cos2\alpha x+\phi_{2}\cos2\beta x+\phi_{3}\sin\alpha x\sin\beta y+\phi_{4}\sin3\alpha x\sin\beta y\\ \;\;\;\;\;\;\;\;\;\;\;\;\;\;\;\;\;\;\;+\phi_{5}\cos\alpha x\cos\beta y+\phi_{6}\cos3\alpha x\cos\beta y-\frac{1}{2}\sigma_{0y}hx^{2}, \end{array}$$

where

$$\begin{array}{l} \phi_{1}=\frac{\beta^{2}}{32\alpha^{2}H_{22}}f_{1}^{2}-\frac{1}{8R\alpha^{2}H_{22}}f_{2},\;\;\;\;\;\;\phi_{2}=\frac{\alpha^{2}}{32\beta^{2}H_{11}}f_{1}^{2},\;\;\;\;\;\;\;\phi_{3}=\frac{f_{1}P_{1}+f_{1}f_{2}P_{2}}{P_{3}},\\ \phi_{4}=\frac{P_{4}}{P_{5}}f_{1}f_{2},\;\;\;\;\;\;\;\;\;\;\phi_{5}=\frac{P_{6}f_{1}+P_{7}f_{1}f_{2}}{P_{3}},\;\;\;\;\;\;\;\;\;\;\;\;\phi_{6}=\frac{P_{8}}{P_{5}}f_{1}f_{2}, \end{array}\frac{m\pi }{L}=\alpha ,\frac{n}{R}=\beta ,\begin{array}{c} P_{1}=\left[\alpha^{4}H_{22}+\alpha^{2}\beta^{2}\left(2H_{12}+H_{66}\right)+\beta^{4}H_{11}\right]\frac{\alpha^{2}}{R},\\ P_{2}=-\alpha^{2}\beta^{2}\left[\alpha^{4}H_{22}+\alpha^{2}\beta^{2}\left(2H_{12}+H_{66}\right)+\beta^{4}H_{11}\right],\\ P_{3}={\left[\alpha^{4}H_{22}+\alpha^{2}\beta^{2}\left(2H_{12}+H_{66}\right)+\beta^{4}H_{11}\right]}^{2}-{\left(2\alpha^{3}\beta H_{26}+2\alpha \beta^{3}H_{16}\right)}^{2},\\ P_{4}=\alpha^{2}\beta^{2}\left[81\alpha^{4}H_{22}+9\alpha^{2}\beta^{2}\left(2H_{12}+H_{66}\right)+\beta^{4}H_{11}\right],\\ P_{5}={\left[81\alpha^{4}H_{22}+9\alpha^{2}\beta^{2}\left(2H_{12}+H_{66}\right)+\beta^{4}H_{11}\right]}^{2}-3{\left(18\alpha^{3}\beta H_{26}+2\alpha \beta^{3}H_{16}\right)}^{2},\\ P_{6}=-\alpha \beta \left(2\alpha^{2}H_{26}+2\beta^{2}H_{16}\right)\frac{\alpha^{2}}{R},\begin{array}{l} P_{7}=\alpha^{3}\beta^{3}\left(2\alpha^{2}H_{26}+2\beta^{2}H_{16}\right),\\ P_{8}=-3\alpha^{3}\beta^{3}\left(18\alpha^{2}H_{26}+2\beta^{2}H_{16}\right). \end{array} \end{array}$$

Substituting the solution form of deflection Equation (18) and stress function form Equation (19) into Equation (14) and then performing the Galerkin procedure in the integral domain 0≤x≤L and 0≤y≤2πR, leads to

(20) $$-K_{1}\left(f_{2}-2f_{0}\right)-2\sigma_{0y}\frac{h}{R}+2q_{0}=0,$$

(21) $$f_{1}\left(Q_{1}+\sigma_{0y}h\beta^{2}\right)+f_{1}f_{2}Q_{2}+f_{1}f_{2}^{2}Q_{3}+f_{1}^{3}Q_{4}=0,$$

(22) $$f_{1}^{2}Q_{5}+f_{1}^{2}f_{2}Q_{6}+f_{2}Q_{7}-\frac{\sigma_{0y}h}{R}+f_{0}K_{1}+q_{0}=0,$$

where

$$\begin{array}{c} Q_{1}=-\frac{P_{1}}{P_{3}}\frac{\alpha^{2}}{R}\;+K_{1}\alpha^{2}+K_{2}\left(\alpha^{2}+\beta^{2}\right)-\left[\alpha^{4}D_{11}+\alpha^{2}\beta^{2}\left(2D_{12}+4D_{66}\right)+\beta^{4}D_{22}\right],\\ Q_{2}=-\frac{P_{2}}{P_{3}}\frac{\alpha^{2}}{R}+\frac{P_{1}}{P_{3}}\alpha^{2}\beta^{2}+\frac{\beta^{2}}{4H_{22}R},Q_{3}=\frac{P_{2}}{P_{3}}\alpha^{2}\beta^{2}-\frac{P_{4}}{P_{5}}\alpha^{2}\beta^{2},\\ Q_{4}=-\frac{1}{16H_{11}}\alpha^{4}-\frac{1}{16H_{22}}\beta^{4},\;\;\;\;\;\;\;Q_{5}=\frac{1}{16H_{22}}\frac{\beta^{2}}{R}+\frac{1}{2}\frac{P_{1}}{P_{3}}\alpha^{2}\beta^{2},\\ Q_{6}=\frac{1}{2}\left(\frac{P_{2}}{P_{3}}-\frac{P_{4}}{P_{5}}\right)\alpha^{2}\beta^{2},\;\;\;\;\;\;\;Q_{7}=-\frac{1}{4H_{22}R^{2}}-4\alpha^{4}D_{11}-\frac{3K_{1}}{4}-K_{2}\alpha^{2}. \end{array}$$

Unlike the cylindrical panel, the circumferential closed condition in an average sense must be satisfied for all revolution shell types, as

(23) $$\int_{0}^{2\pi R}\int_{0}^{L}\left(\varepsilon_{y}^{0}+\frac{w}{R}-\frac{1}{2}w_{,y}^{2}\right)dxdy=0.$$

Using Equations (15) and (18), the closed condition Equation (23) becomes

(24) $$\frac{2f_{0}}{R}+\frac{f_{2}}{R}-2H_{22}\sigma_{0y}h-\frac{\beta^{2}}{4}f_{1}^{2}=0.$$

Eliminating $\sigma_{0y}$ from Equations (20)–(22) and combining them with the condition of a circumferential closed form of shell presented in Equation (24), leads to

(25) $$q_{0}=-f_{2}^{2}\frac{Q_{3}}{U_{2}}+f_{2}\frac{U_{3}}{U_{2}}+\frac{f_{2}}{f_{2}Q_{6}+Q_{5}}\frac{U_{4}}{U_{2}}-\frac{Q_{1}}{U_{2}},$$

where

$$\begin{array}{c} U_{1}=\frac{\beta^{4}R^{2}K_{1}\alpha^{2}h}{8(R^{2}K_{1}H_{22}+1)}-Q_{4}\alpha^{2}h,U_{2}=\beta^{2}R-\frac{n^{2}RK_{1}H_{22}}{\left(R^{2}K_{1}H_{22}+1\right)},\\ U_{3}=\frac{\beta^{2}RK_{1}}{2}-Q_{2}-\frac{n^{2}K_{1}\left(H_{22}RK_{1}+\frac{1}{R}\right)}{2(H_{22}R^{2}K_{1}+1)},U_{4}=Q_{4}(Q_{7}+\frac{K_{1}}{2})-\frac{\beta^{4}R^{2}K_{1}(Q_{7}+\frac{K_{1}}{2})}{8(H_{22}R^{2}K_{1}+1)}. \end{array}$$

When the nonlinear amplitude of the postbuckling state $f_{2}\to 0$, Equation (25) becomes

(26) $$q_{0}^{upper}=-\frac{Q_{1}}{U_{2}},$$

where $q_{0}^{upper}$ is the upper buckling pressure of spiral corrugated sandwich FGM cylindrical shells.

The critical buckling pressure $q_{0}^{cr}$ of spiral corrugated sandwich FGM cylindrical shells is calculated by $q_{0}^{cr}=\min q_{0}^{upper}$ vs. (m,n).

By using Equations (20)–(22), the maximal deflection of a shell located at $x=\frac{L}{2m}i$ and $y=\frac{\pi R}{2n}j$ (with the odd integer numbers i and j) is written by respecting the amplitude of the nonlinear postbuckling state $f_{2}$, as

(27) $$\begin{array}{l} W_{\max}=f_{0}+f_{1}+f_{2}\\ \;\;\;\;\;\;\;\;\;\;\;\;\;=\frac{R}{2(H_{22}R^{2}K_{1}+1)}\left[-\frac{\beta^{2}f_{2}(2Q_{7}+K_{1})}{8(f_{2}Q_{6}+Q_{5})}-f_{2}(H_{22}RK_{1}+\frac{1}{R})+2H_{22}Rq_{0}\right]\\ \;\;\;\;\;\;\;\;\;\;\;\;\;\;\;\;+{\left[-\frac{f_{2}(Q_{7}+\frac{K_{1}}{2})}{f_{2}Q_{6}+Q_{5}}\right]}^{\frac{1}{2}}+f_{2}. \end{array}$$

The postbuckling curves of spiral corrugated sandwich FGM cylindrical shells can be determined by combining Equation (25) and Equation (27).

## 5. Numerical Results and Remarks

In this section, the accuracy of the semi-analytical approach presented for the critical buckling pressure of isotropic cylindrical shells and sandwich FGM cylindrical shells under external pressure is compared with the results reported by Shen et al. [5], Vuong and Duc [15] using higher-order shear deformation shell theory, and Nam et al. [18] using Donnell shell theory. The postbuckling results are compared with the results of non-corrugated ceramic and metal cylindrical shells reported by Dung and Hoa [16].

**Figure 2:**
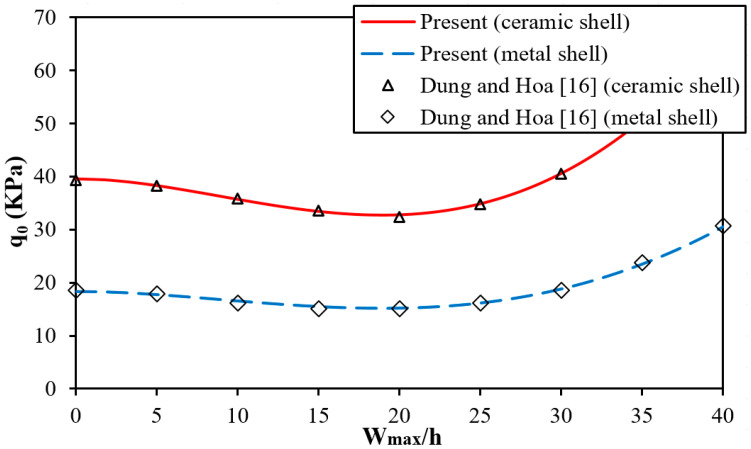
Comparison of postbuckling curves of ceramic and metal cylindrical shells and the results of Dung and Hoa [16] (h=0.305 mm, R=60.643 mm, L=387.35 mm, ν=0.3, and E=151 GPa for a ceramic shell, and E=70 GPa for a metal shell).

As can be seen in Table 1 and Table 2 and Figure 2, there are very small differences between the present results and previous results.

Numerical investigations were performed to predict the nonlinear buckling responses of spiral corrugated sandwich FGM cylindrical shells subjected to external pressure and surrounded by an elastic medium. The sandwich FGM corrugated cylindrical shells with the following geometrical parameters were considered: L= 0.75 m, R= 0.5 m, h= mm, and $h_{f}=h/4$. The results of the corrugated sandwich FGM cylindrical shells were compared with those of corresponding non-corrugated sandwich FGM cylindrical shells in the case of the same material quantity. The volume of material of the non-corrugated shells was equal to that of corresponding corrugated shells, if $h^{Non-Corrugated}=\frac{l}{e}h^{Non-Corrugated},h_{c}^{Non-Corrugated}=\frac{l}{e}h_{c}^{Corrugated}.$ The combination of materials was Aluminum $E_{m}=7\times 10^{10}$ N/m^2^ and Alumina $E_{c}=38\times 10^{10}$ N/m^2^, and the Poisson ratio was assumed to be a constant value of ν=0.3.

Table 3 shows the critical buckling pressure $q_{0}^{cr}$ for non-corrugated, ring corrugated, and spiral corrugated sandwich FGM cylindrical shells with different volume fraction indexes and numbers of corrugation waves in the loop coil. To evaluate the corrugation effect, the thickness of the non-corrugated sandwich FGM cylindrical shell was determined so that the quantities of material in the two non-corrugated and corrugated shell cases were equal. Clearly, the critical buckling pressure of a ring corrugated sandwich FGM cylindrical shell is significantly greater than that of non-corrugated sandwich FGM cylindrical shells. In addition, the effect of spiral corrugation on the critical buckling pressure of shells is especially great. When the number of corrugation waves increases, the critical buckling pressure increases and it reaches a maximal value at a very high number of corrugation waves in a loop coil. The effect of the volume fraction index is very large with non-corrugated shells; on the contrary, this effect decreases in the case of corrugated shells and is insignificant for the maximal value of critical pressure.

Table 4 and Table 5 present the effect of geometrical parameters of the cross section of corrugation on the critical buckling pressure of corrugated sandwich FGM cylindrical shells with round and trapezoidal forms, respectively. Clearly, the geometrical parameters of the cross section of corrugations are strongly influenced by the critical pressure of shells. In addition, the effects of geometrical properties of corrugation are complex and different with round and trapezoidal corrugated shells.

The effect of elastic foundation moduli is presented in Table 6 and Table 7 for round and trapezoidal corrugated shells, respectively. It is obvious that the critical buckling pressure of corrugated shells is greater with the support of an elastic foundation and the critical buckling pressure increases if the elastic foundation moduli increase.

**Figure 3:**
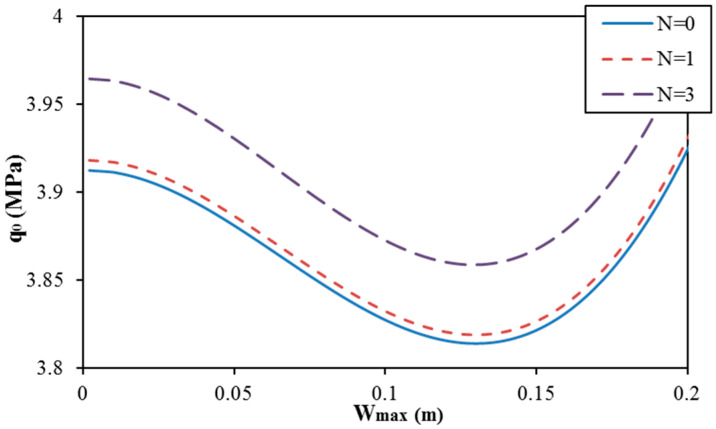
Effect of the number of waves of corrugation in a loop coil on the postbuckling curve of round spiral corrugated sandwich FGM cylindrical shells ($K_{1}$ = 10^7^ N/m^3^; $K_{2}$ = 5 × 10^4^ N/m, d = 0, r = 0.015 m, and k = 1).

The postbuckling curves of round spiral corrugated sandwich FGM cylindrical shells are shown in Figure 3. Spiral corrugation with the number of waves of corrugation in a loop coil of N=0 (ring corrugation) and N=1,  N=3 (spiral corrugation) is considered. It seems that the number of waves of corrugation in a loop coil strongly influences the postbuckling behavior of cylindrical shells, and the usual tendencies are observed with various numbers of waves of corrugation in a loop coil.

**Figure 4:**
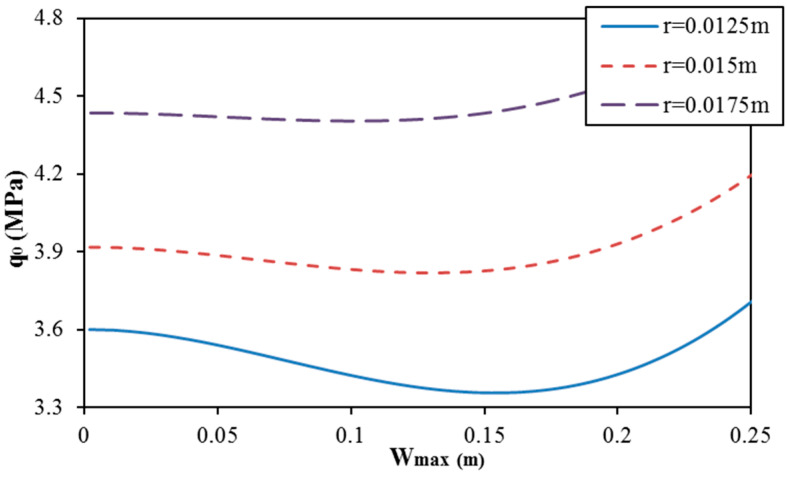
Effect of the corrugation radius r on the postbuckling curve of round spiral corrugated sandwich FGM cylindrical shells ($K_{1}$ = 10^7^ N/m^3^, $K_{2}$ = 5 × 10^4^ N/m, N = 1, d = 0, and k = 1).

**Figure 5:**
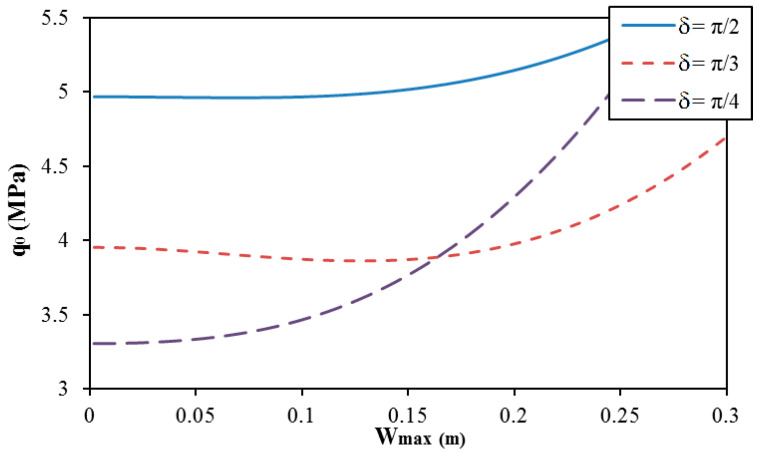
Effect of the angle δ of corrugation on the postbuckling curve of trapezoidal spiral corrugated sandwich FGM cylindrical shells ($K_{1}$ = 10^7^ N/m^3^, $K_{2}$ = 5 × 10^4^ N/m, N =1; e = 0.03 m, and k = 1).

The effect of geometrical parameters of corrugation (r for round corrugation and δ for trapezoidal corrugation) on the postbuckling curve of spiral corrugated sandwich FGM cylindrical shells with round and trapezoidal corrugation cases is presented in Figure 4 and Figure 5. The results show that the geometrical parameters of corrugation strongly influence the postbuckling curve of cylindrical shells. The complex behavior and unusual tendencies are obtained with different geometrical parameters of corrugations in both cases of round and trapezoidal corrugations.

**Figure 6:**
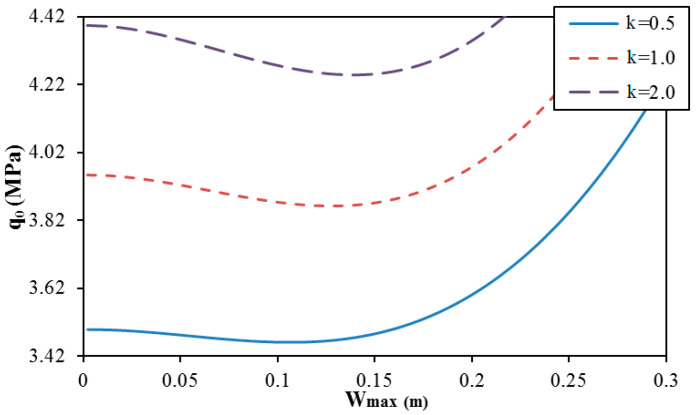
Effect of the volume fraction index k on the postbuckling curve of trapezoidal spiral corrugated sandwich FGM cylindrical shells ($K_{1}$ = 10^7^ N/m^3^, $K_{2}$ = 5 × 10^4^ N/m, N = 1, e = 0.03 m, δ=π/3, and k = 1).

Figure 6 presents the effect of the volume fraction index k of sandwich FGM on the postbuckling curve of trapezoidal spiral corrugated cylindrical shells N = 1). The results show that an increase of the volume fraction index k of sandwich FGM yields a significant increase of the critical buckling pressure and postbuckling strength.

**Figure 7:**
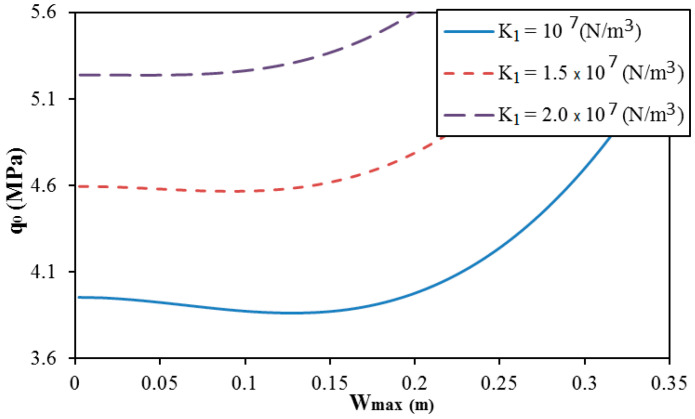
Effect of the Winkler modulus of the elastic foundation $K_{1}$ on the postbuckling curve of trapezoidal spiral corrugated sandwich FGM cylindrical shells (e=0.03m, f=0.015m, δ=π/3,  k=1, N = 1, and $K_{2}=5\times 10^{4}$ N/m).

**Figure 8:**
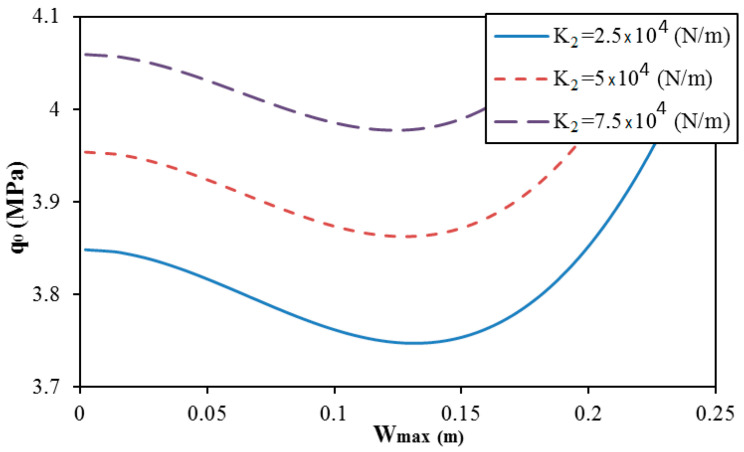
Effect of the shear layer modulus of the elastic foundation $K_{2}$ on the postbuckling curve of trapezoidal spiral corrugated sandwich FGM cylindrical shells (e=0.03m, f=0.015m, δ=π/3,  k=1, N = 1, and $K_{1}=10^{7}$ N/m^3^).

The effect of elastic foundation moduli on the postbuckling strength of trapezoidal corrugated sandwich FGM cylindrical shells is presented in Figure 7 and Figure 8. The elastic foundation moduli increase yields an increase of the postbuckling strength of cylindrical shells. It seems that the snap-through phenomenon insignificantly varies when the shear layer modulus of the elastic foundation changes. On the contrary, the snap-through phenomenon is reduced when the Winkler modulus of the elastic foundation increases.

## 6. Conclusions

In this paper, the nonlinear buckling and postbuckling behavior of spiral corrugated sandwich FGM cylindrical shells under external pressure and surrounded by an elastic medium has been formulated by applying the homogenization model of corrugated panels and the coordinate transformation procedure. The results validated the large effects of corrugation on the buckling and postbuckling behavior of shells.

Some significant results have been obtained as follows:

(1) Corrugated sandwich FGM shells are homogenized to non-corrugated sandwich FGM shells;
(2) The critical buckling pressure of a corrugated sandwich FGM cylindrical shell is greater than that of a corresponding non-corrugated sandwich FGM cylindrical shell;
(3) The geometrical parameters of the cross section of corrugation and the material properties strongly influence the buckling and postbuckling behavior of sandwich FGM cylindrical shells.

## Figures and Tables

**Table 1:** Comparison of the upper critical buckling pressure (kPa) for an isotropic cylindrical shell under external pressure and previous results ($L=\sqrt{RhZ}$, R=0.4 m, Z=500, E=323.11 GPa, and ν=0.3, without an elastic foundation).

R/h	Shen et al. [5]	Vuong and Duc [15]	Present
40	9112.24 (1, 4) *	9237.57 (1, 4)	9232.14 (1, 4)
400	87.4899 (1, 11)	88.5202 (1, 11)	87.9851 (1, 11)

* The numbers in parentheses denote the buckling mode (m,n).

**Table 2:** Comparison of the upper critical buckling pressure (MPa) of a sandwich FGM cylindrical shell and previous results (h=5 mm, R/h=100, L/R=2, $h_{f}=2$ mm, $E_{c}=321.87$ GPa, and $E_{m}=207.76$ GPa).

Studies	k=0.2	k=1	k=2	k=10
Nam et al. [18]	1.1474 (1, 6)	1.3672 (1, 6)	1.4519 (1, 6)	1.5334 (1, 6)
Present	1.1474 (1, 6)	1.3672 (1, 6)	1.4519 (1, 6)	1.5334 (1, 6)

**Table 3:** The critical buckling pressures of non-corrugated and round corrugated sandwich FGM cylindrical shells (MPa) ($K_{1}=10^{7}N/m^{3},K_{2}=5\times 10^{4}N/m,\;r=0.015m;\;\;d=0.005m$).

k	Non-corrugated	Ring Corrugated	Spiral Corrugated				
			N = 1	N = 3	N = 5	N = 10	Maximal
0.1	0.533 (1, 8)	3.803 (1, 2)	3.807 (1, 2)	3.842 (1, 2)	3.914 (1, 2)	4.304 (1, 2)	29.272 (1, 5, 44) **
0.5	0.776 (1, 7)	4.879 (1, 2)	4.886 (1, 2)	4.937 (1, 2)	5.042 (1, 2)	5.574 (1, 2)	30.423 (1, 5 , 43)
1	0.922 (1, 7)	5.613 (1, 2)	5.621 (1, 2)	5.683 (1, 2)	5.810 (1, 2)	6.445 (1, 2)	30.783 (1, 4, 42)
2	1.054 (1, 7)	6.340 (1, 2)	6.349 (1, 2)	6.422 (1, 2)	6.571 (1, 2)	7.308 (1, 2)	31.983 (1, 4, 42)
10	1.210 (1, 7)	7.377 (1, 2)	7.388 (1, 2)	7.477 (1, 2)	7.656 (1, 2)	8.536 (1, 2)	34.796 (1, 4, 42)

** The numbers in parentheses denote the buckling mode and number of waves of corrugation in a loop coil (m,n,N).

**Table 4:** The critical buckling pressure of corrugated sandwich FGM cylindrical shells with different values of geometrical parameters of round corrugation (MPa) ($K_{1}=10^{7}N/m^{3},$ $K_{2}=5\times 10^{4}\;N/m,k=1$).

r	d	Non-corrugated	Ring Corrugated	Spiral Corrugated			
				N = 1	N = 3	N = 5	Maximal
0.0125	0	0.638 (1, 8)	3.597 (1, 2)	3.601 (1, 2)	3.6336 (1, 2)	3.701 (1, 2)	6.478 (1, 5, 47)
	0.01	1.475 (1, 7)	7.472 (1, 2)	7.479 (1, 2)	7.5385 (1, 2)	7.659 (1, 2)	61.491 (1, 5, 52)
0.015	0	0.638 (1, 8)	3.912 (1, 2)	3.918 (1, 2)	3.9644 (1, 2)	4.059 (1, 2)	10.303 (1, 4, 40)
	0.01	1.294 (1, 7)	8.481 (1, 2)	8.494 (1, 2)	8.5922 (1, 2)	8.793 (1, 2)	81.550 (1, 5, 44)
0.0175	0	0.638 (1, 8)	4.427 (1, 2)	4.435 (1, 2)	4.5031 (1, 2)	4.643 (1, 2)	15.805 (1, 5, 36)
	0.01	1.176 (1, 7)	9.612 (1, 2)	9.631 (1, 2)	9.7860 (1, 2)	10.105 (1, 2)	107.335 (1, 5, 38)

**Table 5:** The critical buckling pressure of corrugated sandwich FGM cylindrical shells with different values of geometrical parameters of trapezoidal corrugation (MPa) ($K_{1}=10^{7}N/m^{3},$ $K_{2}=5\times 10^{4}\;N/m,\;\;f=0.015m,\;\;k=1$).

δ	e	Non-corrugated	Ring Corrugated	Spiral Corrugated				
				N = 1	N = 3	N = 5	Maximal	
$\frac{\pi }{4}$	0.025	0.582 (1, 8)	2.769 (1, 3)	2.769 (1, 3)	2.774 (1, 3)	2.785 (1, 3)	4.181 (1, 4, 44)	
	0.035	0.490 (1, 8)	3.602 (1, 2)	3.610 (1, 2)	3.675 (1, 2)	3.734 (1, 3)	6.467 (1, 4, 33)	
$\frac{\pi }{3}$	0.025	0.740 (1, 7)	3.884 (1, 2)	3.888 (1, 2)	3.920 (1, 2)	3.985 (1, 2)	10.029 (1, 5, 48)	
	0.035	0.581 (1, 8)	3.996 (1, 2)	4.004 (1, 2)	4.067 (1, 2)	4.199 (1, 2)	11.153 (1, 5, 35)	
$\frac{\pi }{2}$	0.025	1.247 (1, 7)	5.099 (1, 2)	5.104 (1, 2)	5.144 (1, 2)	5.223 (1, 2)	24.080 (1, 5, 50)	
	0.035	0.878 (1, 7)	4.862 (1, 2)	4.871 (1, 2)	4.945 (1, 2)	5.096 (1, 2)	20.999 (1, 5, 36)	

**Table 6:** The critical buckling pressure of round corrugated sandwich FGM cylindrical shells with different values of elastic foundation moduli (MPa) (r=0.015m,d=0.005m,k=1).

$K_{1}\;(N/m^{3})$	$K_{2}\;(N/m)$	Non-corrugated	Ring Corrugated	Spiral Corrugated			
				N = 1	N = 3	N = 5	N = 10
0	0	0.707 (1, 7)	4.129 (1, 2)	4.136 (1, 2)	4.198 (1, 2)	4.321 (1, 2)	4.905 (1, 2)
10^7^	0	0.813 (1, 7)	5.403 (1, 2)	5.410 (1, 2)	5.473 (1, 2)	5.599 (1, 2)	6.233 (1, 2)
	5 × 10^4^	0.922 (1, 7)	5.613 (1, 2)	5.621 (1, 2)	5.683 (1, 2)	5.810 (1, 2)	6.445 (1, 2)
1.5 × 10^7^	0	0.866 (1, 7)	6.044 (1, 2)	6.052 (1, 2)	6.115 (1, 2)	6.244 (1, 2)	6.909 (1, 2)
	5 × 10^4^	0.976 (1, 7)	6.255 (1, 2)	6.263 (1, 2)	6.326 (1, 2)	6.455 (1, 2)	7.122 (1, 2)
2 × 10^7^	0	0.919 (1, 7)	6.688 (1, 2)	6.696 (1, 2)	6.759 (1, 2)	6.891 (1, 2)	7.592 (1, 2)
	5 × 10^4^	1.029 (1, 7)	6.899 (1, 2)	6.907 (1, 2)	6.971 (1, 2)	7.103 (1, 2)	7.807 (1, 2)

**Table 7:** The critical buckling pressure of trapezoidal corrugated sandwich FGM cylindrical shells with different values of elastic foundation moduli (MPa) (e=0.03m, f=0.015m,δ=π/3,  k=1).

$K_{1}\;(N/m^{3})$	$K_{2}\;(N/m)$	Non-corrugated	Ring Corrugated	Spiral Corrugated			
				N = 1	N = 3	N = 5	N = 10
0	0	0.442 (1, 7)	2.467 (1, 2)	2.473 (1, 2)	2.519 (1, 2)	2.612 (1, 2)	3.086 (1, 2)
10^7^	0	0.535 (1, 8)	3.737 (1, 2)	3.743 (1, 2)	3.789 (1, 2)	3.884 (1, 2)	4.378 (1, 2)
	5 × 10^4^	0.643 (1, 8)	3.948 (1, 2)	3.954 (1, 2)	4.000 (1, 2)	4.095 (1, 2)	4.590 (1, 2)
1.5 × 10^7^	0	0.576 (1, 8)	4.377 (1, 2)	4.383 (1, 2)	4.430 (1, 2)	4.526 (1, 2)	5.033 (1, 2)
	5 × 10^4^	0.684 (1, 8)	4.589 (1, 2)	4.594 (1, 2)	4.641 (1, 2)	4.738 (1, 2)	5.246 (1, 2)
2 × 10^7^	0	0.617 (1, 8)	5.021 (1, 2)	5.027 (1, 2)	5.074 (1, 2)	5.171 (1, 2)	5.680 (1, 3)
	5 × 10^4^	0.725 (1, 8)	5.233 (1, 2)	5.238 (1, 2)	5.286 (1, 2)	5.383 (1, 2)	5.832 (1, 3)
